# Substrate Stiffness Influences Structural and Functional Remodeling in Induced Pluripotent Stem Cell-Derived Cardiomyocytes

**DOI:** 10.3389/fphys.2021.710619

**Published:** 2021-08-19

**Authors:** Arlene Körner, Matias Mosqueira, Markus Hecker, Nina D. Ullrich

**Affiliations:** ^1^Division of Cardiovascular Physiology, Institute of Physiology and Pathophysiology, Heidelberg University, Heidelberg, Germany; ^2^German Center for Cardiovascular Research (DZHK), Partner Site Heidelberg-Mannheim, Heidelberg, Germany

**Keywords:** induced pluripotent stem cell-derived cardiomyocytes, excitation-contraction coupling, contraction, gap junctions, stiffness

## Abstract

Novel treatment strategies for cardiac tissue regeneration are heading for the use of engineered cardiac tissue made from induced pluripotent stem cell-derived cardiomyocytes (iPSC-CMs). Despite the proven cardiogenic phenotype of these cells, a significant lack of structural and functional properties of mature myocytes prevents safe integration into the diseased heart. To date, maturation processes of cardiomyocytes remain largely unknown but may comprise biophysical cues from the immediate cell environment. Mechanosensing is one critical ability of cells to react to environmental changes. Accordingly, the surrounding substrate stiffness, comprised of extracellular matrix (ECM), cells, and growth surface, critically influences the myocyte’s physiology, as known from deleterious remodeling processes in fibrotic hearts. Conversely, the mechanical properties during culture of iPSC-CMs may impact on their structural and functional maturation. Here, we tested the hypothesis that the environmental stiffness influences structural and functional properties of iPSC-CMs and investigated the effect of different substrate stiffnesses on cell contractility, excitation-contraction (EC) coupling, and intercellular coupling. Culture surfaces with defined stiffnesses ranging from rigid glass with 25GPa to PDMS of physiological softness were coated with ECM proteins and seeded with murine iPSC-CMs. Using confocal imaging, cardiac protein expression was assessed. Ca^2+^ handling and contractile properties were analyzed on different substrate stiffnesses. Intercellular coupling *via* gap junctions was investigated by fluorescence recovery after photobleaching (FRAP). Our data revealed greater organization of L-type Ca^2+^ channels and ryanodine receptors and increased EC-coupling gain, demonstrating structural and functional maturation in cells grown on soft surfaces. In addition, increased shortening and altered contraction dynamics revealed increased myofilament Ca^2+^ sensitivity in phase-plane loops. Moreover, connexin 43 expression was significantly increased in iPSC-CMs grown on soft surfaces leading to improved intercellular coupling. Taken together, our results demonstrate that soft surfaces with stiffnesses in the physiological range improve the expression pattern and interaction of cardiac proteins relevant for EC-coupling. In parallel, soft substrates influence contractile properties and improve intercellular coupling in iPSC-CMs. We conclude that the mechanical stiffness of the cell environment plays an important role in driving iPSC-CMs toward further maturation by inducing adaptive responses.

## Introduction

Cardiovascular medicine is presently facing a new challenge with rapidly rising numbers of patients suffering from heart disease, the leading cause of death worldwide (Virani et al., 2020). Because of the limited regenerative potential of the adult heart, dying cardiomyocytes are replaced by non-contractile connective tissue thereby inducing detrimental structural remodeling (Jiang et al., 2018). More precisely, massive fibroblast proliferation and collagen deposition lead to the formation of a firm scar (Pfeffer and Braunwald, 1990). Remodeling of extracellular matrix (ECM) finally results in fibrosis involving increased stiffness of the cardiac environment (30–55kPa instead of 10–15kPa of healthy cardiac tissue) and leading to impaired contractility (Gaetani et al., 2020). Since therapeutic options for heart failure patients are limited, research in cardiac regenerative medicine is heading toward the generation of engineered human myocardium (EHM) made of induced pluripotent stem cell-derived cardiomyocytes (iPSC-CMs; Fujita and Zimmermann, 2018). Over the last years, reliable differentiation protocols have been established and successful production of iPSC-CMs with an adequately high yield for clinical applications has turned an initial research tool into a realistic option for myocardial repair (Burridge et al., 2012). The electrophysiological profile and contractile activity of iPSC-CMs confirm true cardiogenic features and fuel hope for the development of novel and promising treatment strategies (Silbernagel et al., 2020). Indeed, remuscularization by iPSC-CMs grafts and improvement of cardiac function after myocardial infarction have already been demonstrated in various animal models (Chong et al., 2014; Gao et al., 2018; Pecha et al., 2019).

Nevertheless, iPSC-CMs still present an immature and variable phenotype with functional features similar to cardiomyocytes of early developmental stages (Yang et al., 2014). In contrast to the elongated anisotropic shape and precise microarchitecture of adult cardiomyocytes, iPSC-CMs show an irregular and unspecific geometry resulting in a diffuse intracellular distribution of myofibrils with variable degrees of sarcomere organization (Gherghiceanu et al., 2011; Silbernagel et al., 2020). Another ultrastructural deficiency in iPSC-CMs is given by the lack of transverse (t)-tubules (Yang et al., 2014). In the adult heart, these highly specialized sarcolemmal structures come in close vicinity to the end-cisterns of the sarcoplasmic reticulum (SR) spaced by a 15 to 20nm wide dyadic cleft. This special arrangement allows for close interaction of the sarcolemmal L-type Ca^2+^ channels (LTCCs) and the ryanodine receptors (RyR2s), the Ca^2+^ release channels of the SR. Both channels are fundamental to the mechanism of excitation-contraction (EC) coupling and control cytosolic Ca^2+^ entry by Ca^2+^-induced Ca^2+^ release (CICR), which links electrical excitation to contractile activity and force production by the myocyte in a highly spatiotemporally synchronized way (Bers, 2002; Kane et al., 2015). Although CICR has been demonstrated in iPSC-CMs (Itzhaki et al., 2011), these cells show immature Ca^2+^ handling and desynchronized Ca^2+^ transients (Lieu et al., 2009). The spontaneous contractile activity of iPSC-CMs is another hallmark of immaturity, which is probably caused by spontaneous Ca^2+^ release from the SR triggering depolarizing membrane current *via* the sodium-calcium-exchanger (NCX; Zahanich et al., 2011; Kane et al., 2015; Karbassi et al., 2020).

The consequence of missing t-tubules and thus poor coupling of LTCCs and RyR2s is inefficient Ca^2+^ handling, which may lead to enhanced activity of the so-called orphaned RyR2s (Lee et al., 2011; Rao et al., 2013). Moreover, in addition to inefficient EC-coupling, we have recently described weaknesses in intercellular coupling between iPSC-CMs. As iPSC-CMs do not develop a precise structural orientation, intercalated disks are not well-defined with the consequence of a diffuse distribution of gap junctions and their main subunit connexin-43 (Cx43). Since the expression pattern of Cx43 is not localized at specific end poles of the cell as known from adult cardiomyocytes, electrical signal propagation is not directed but diffuse and heterogeneous across the cell layer. In addition, reduced clustering of Cx43 results in significantly slower conduction velocity compared to native cardiomyocytes (Kucera et al., 2015; Marcu et al., 2015; Jiang et al., 2018; Sottas et al., 2018; Karbassi et al., 2020).

In light of future therapeutic applications, immature Ca^2+^ handling and reduced intercellular coupling in iPSC-CMs present high-risk factors for the development of arrhythmogenic modifications after implantation (Shiba et al., 2012; Chong et al., 2014). To improve the electrophysiological properties of iPSC-CMs, different approaches have been tested to enhance functional maturation in iPSC-CMs at the level of EC-coupling. They include natural time-dependent maturation processes during long-term culture, the addition of hormones, such as triiodothyronine (Kamakura et al., 2013; Lundy et al., 2013; Yang et al., 2014), or enhanced expression of Cx43 (Sottas et al., 2018). Moreover, co-cultures with non-cardiomyocytes were shown to promote maturity features (Kroll et al., 2017; Yoshida et al., 2018), which may also explain the successful transplantation experiments of iPSC-CMs into adult hearts providing a more natural cell environment compared to culture conditions (Kadota et al., 2017). Another important aspect of the cardiac environment influencing maturation is the composition of the ECM (Young et al., 2014). The combination of iPSC-CM growth together with non-cardiomyocytes in a specifically composed ECM led to the development of 3D-engineered human myocardium, which improved cardiac properties after implantation in animal models (Tiburcy et al., 2017; Weinberger et al., 2017; Ronaldson-Bouchard et al., 2018; Yeung et al., 2019).

Furthermore, the stiffness of the surrounding material is another critical parameter of the natural cardiac environment, and its influence on the functional improvement and maturation of EC-coupling in iPSC-CMs has not been elucidated yet. Tissue stiffness changes significantly during cardiac development from the fetal to the adult heart, and conversely, structural remodeling in the diseased heart, such as inflammation and fibrotic lesions, leads to further changes in cardiac tissue stiffness. Altered protein expression, including the expression of different isoforms of integrin receptors, may control the sensing and interaction of the cell with its environment and trigger outside-in signaling pathways, which induce long-term remodeling processes influencing structural and functional properties of the cardiomyocytes (Ward and Iskratsch, 2020). Extrapolated to the development of iPSC-CMs, the stiffness of the growth surface and immediate environment may have a strong impact on further maturation processes in these young cardiomyocytes.

In this study, we tested the hypothesis that substrate stiffness of the cell environment influences the structural and functional maturation of iPSC-CMs. Cells were grown on different culture surfaces with defined stiffnesses ranging from rigid glass (25GPa) to the silicone-based organic polymer polydimethylsiloxane (PDMS) of different softness (28kPa, 15kPa, and 1.5kPa). We focused on intracellular Ca^2+^ handling and analyzed EC-coupling properties with a special focus on the expression and function of the LTCC and RyR2. Moreover, we investigated Cx43 expression in iPSC-CMs grown on soft surfaces and evaluated intercellular communication in cell monolayers. We demonstrate that iPSC-CMs grown on surfaces of physiological stiffness exhibit more mature structural and functional properties at the level of Ca^2+^ handling and intercellular coupling compared to cells grown on rigid surfaces. Thus, specific control of the environmental properties increases the potential of iPSC-CMs to develop into mature cardiomyocytes that can be used for cardiac engineering and cell replacement therapies for diseased hearts.

## Materials and Methods

### Cell Models

Murine iPSC-CMs were received from Ncardia (Cologne, Germany) and kept in liquid nitrogen until use. Culture dishes were coated with a mixture of laminin and fibronectin in PBS (1:1:100) overnight at 37°C to enable attachment of iPSC-CMs. After defrosting, cells were seeded in Cor.AT^®^ medium (Ncardia, Cologne, Germany) at a density of 2×10^4^ cells per dish for Ca^2+^ measurements and on glass coverslips in 24-well plates (Sarstedt, Nümbrecht, Germany) for immunostainings, and at 10^4^ cells per dish onto glass-bottom dishes (35mm, MatTek, Ashland, MA, United States) and PDMS-coated dishes (35mm, Ibidi GmbH, Gräfelfing, Germany) for electrophysiological experiments and live-cell imaging analysis.

To avoid the growth of undifferentiated cells and non-cardiomyocytes, the cardiomyocyte-specific α-myosin heavy chain (α-MHC) promoter was chosen to control pac gene expression for puromycin resistance. Puromycin (1μg/ml) was added for the selection of cardiac-specific cells for the first 48h in culture. Afterward, cells were kept in culture in puromycin-free Cor.At^®^ medium. Cells were maintained in culture at 37°C and 5% CO_2_ and used within 4weeks.

### Live-Cell Imaging and Cellular Electrophysiology

For all live-cell imaging experiments, the standard bath solution was cardiac Tyrode’s solution containing (in mm): NaCl 140, KCl 5.4, CaCl_2_ 1.8, MgCl_2_ 1.1, HEPES 5, glucose 10, pH 7.4.

#### Measurement of Ca^2+^ Transients and Myocyte Contractility

Ca^2+^ transients of iPSC-CMs were recorded using the ratiometric Ca^2+^-sensitive fluorescent indicator fura-2AM (Thermo Fisher Scientific, Dreieich, Germany). Cells were loaded with 1.5μm fura-2AM diluted in Tyrode’s solution and incubated for 20min, followed by 10min of de-esterification. iPSC-CMs were constantly perfused with prewarmed Tyrode’s solution containing Probenecid (100μm) to avoid sequestration or secretion of fura-2. Using the IonOptix system (IonOptix, Dublin, Ireland), Ca^2+^ transients were recorded in parallel with edge detection to measure contractions. Data were collected by using the IonWizard software developed by IonOptix. Cells were exposed to light emitted by a xenon lamp passing through rapidly switching filters of 340nm and 360nm to determine the ratio of bound and unbound Ca^2+^ ions in the cells. Fluorescence emission light was collected at 510nm. Data are presented as fura-2 ratio (F_340_/F_360_).

For functional evaluation of spontaneous activity, only rhythmically beating iPSC-CMs were used. Five representative Ca^2+^ transients and contractions at steady state were analyzed per cell using OriginPro^®^ software (OriginLab Corporation, Northampton, MA, United States). Assessed parameters comprised peak Ca^2+^ transients and shortening amplitudes, time-to-peak (TTP), full duration at half maximum (FDHM), decay, and frequency of spontaneous activity. Decay of contractions was fitted with a Boltzmann function, whereas decay of Ca^2+^ transients was calculated by an exponential decay function. Diastolic Ca^2+^ levels of paced iPSC-CMs were only evaluated if cells responded to the frequency of electrical stimulation at 1Hz and 2Hz (10V) by a field stimulator (Myopacer, IonOptix, Dublin, Ireland).

#### Electrophysiology With Simultaneous Ca^2+^ Imaging

For investigation of the EC-coupling mechanism, Ca^2+^ currents (*I*_CaL_) were measured in iPSC-CMs *via* the patch-clamp technique with simultaneous recording of Ca^2+^ signals by confocal line-scan imaging. Experiments were performed using a HEKA EPC-10 patch-clamp amplifier (HEKA Elektronik GmbH, Reutlingen, Germany) connected to an Olympus IX81 laser scanning confocal microscope (Olympus Fluoview FV1000, Olympus, Hamburg, Germany). For patch-clamp recordings, borosilicate glass pipettes were pulled to obtain tip resistances of 2–9 MΩ and filled with an internal solution containing 8mm NaCl, 120mm CsAsp, 20mm TEA-Cl, 5.9mm MgCl_2_, 20mm HEPES, 5mmK_2_-ATP, and 50μm of the Ca^2+^-sensitive fluorescent indicator K_5_-fluo-3 (Thermo Fisher Scientific, Dreieich, Germany). For *I*_CaL_ measurements, iPSC-CMs were constantly perfused with warm Tyrode’s solution (37°C) containing 5mm CsCl.

For analysis of the EC-coupling gain, a two-step protocol was applied (adapted from Ullrich et al., 2012). After inactivation of voltage-dependent Na^+^ channels by a 500ms ramp ranging from the holding potential of −80mv to −40mv, the first test step was set to −25mv for 400ms, followed by a second 400ms test step from −40mv to +10mv to record maximal *I*_CaL_ and Ca^2+^ release amplitudes.

For line-scan imaging, the excitation wavelength was set at 473nm and fluorescence emission was collected between 490nm and 545nm. Line-scan images were recorded at 2μs/pixel, 2ms/line, and 4,000 lines/image. ImageJ/Fiji software was used for image analysis. The fluorescence intensity of line-scan images was plotted over time. The background was subtracted, and line profiles were normalized to baseline. Data are displayed as F/F_0_.

For analysis of the EC-coupling gain, the ratio of the peak Ca^2+^ transient amplitude at −25mv and corresponding peak *I*_CaL_ amplitude was calculated. Cells with T-type Ca^2+^ current at −25mv (high amplitude and fast inactivation) and/or cells with low fluo-3 loading (F/F_0_<1.2) were excluded from analysis. As a control, the ratio of the maximal Ca^2+^ transient and current amplitudes at +10mv was calculated. *I*_CaL_ at +10mv was used for the measurement of maximal current amplitude and inactivation kinetics. Fitting *I*_CaL_ with a biexponential function in OriginPro allowed the comparison of tau_1_ (τ_1_) as an indicator of Ca^2+^-dependent inactivation.

For examination of fractional release and NCX activity in iPSC-CMs, patched cells were stimulated to steady-state activity at a frequency of 1Hz, followed by application of the RyR2 agonist caffeine (10mm in Tyrode’s solution, Sigma-Aldrich) for total depletion of the SR. In parallel with the measurement of SR Ca^2+^ content, caffeine-elicited NCX currents were recorded.

#### Fluorescence Recovery After Photobleaching

For functional evaluation of gap junctions, fluorescence recovery after photobleaching (FRAP) was measured using the gap junction-permeant dye calcein (0.5μm calcein-AM, Thermo Fisher Scientific, Dreieich, Germany). Photobleaching and imaging were done on the Olympus FluoView LSCM using a 60x water immersion objective (1.2 NA). iPSC-CMs were loaded with 0.5μm calcein-AM in Tyrode’s solution for 20min. After 10min of de-esterification in Tyrode’s solution, calcein-diffusion dynamics between iPSC-CMs were assessed as an indicator of functional Cx43 expression and gap junction formation. One cell in a cluster was bleached with a laser power of 50% at 10μs/pixel for 5s, and fluorescence recovery was recorded in 52 images taken every 10s with a laser power of 0.5–1.5% at 2μs/pixel. Importantly, the target cell had to be fully surrounded by neighboring cells (in 2D), thus representing one building block in a conductive and connected cell layer. Further analysis was done in ImageJ to plot the time course of fluorescence recovery of the bleached cell. After subtraction of background and bleaching point, the graph was normalized to the initial value of fluorescence intensity (before bleaching). The final traces were fitted in OriginPro software with a biexponential function to investigate the fast time constant 1 (τ1) as an indicator of diffusion rate.

### Immunocytochemistry and Image Analysis

For immunostainings, cells were washed with PBS, fixed with 4% paraformaldehyde (Thermo Fisher Scientific, Dreieich, Germany) for 20min, and washed three times with PBS for 10min. Different time points were chosen to examine the influence of growth duration on the expression pattern and spatial organization of different Ca^2+^ handling proteins. Cells grown on PDMS-coated dishes were fixed at days 10, 20, and 25 after seeding, cells on glass coverslips after 9, 13, and 20days. To block unspecific binding sites, cells were incubated with bovine serum albumin dissolved in PBS (10mg/ml, Sigma-Aldrich, Germany) for 0.5h at room temperature. For permeabilization of iPSC-CMs, the blocking solution contained 0.1% Triton X-100. Cells were incubated with primary antibodies against Cx43 (mouse, monoclonal, 1:500, MAB3067, MerckMillipore, Darmstadt, Germany), SERCA (mouse, monoclonal, 1:200, ab2861, MerckMillipore, Darmstadt, Germany), NCX1 (mouse, monoclonal, 1:200, MA3-926, Thermo Fisher Scientific, Waltham, MA, United States) or RyR2 (mouse, monoclonal, 1:200, ab2861, AbCam, Cambridge, MA, United States), and Ca_v_1.2 (rabbit, polyclonal, 1:200, AB10515, MerckMillipore, Darmstadt, Germany) at room temperature for 90min. After three times washing with PBS for 10min, cells were incubated with appropriate secondary antibodies conjugated to Alexa Fluor dyes (1:500, Thermo Fisher Scientific) with different excitation-emission spectra for 1h. After three times washing with PBS for 10min, samples were incubated for 1h in Phalloidin-TRITC (1:2000, P1951-1MG, Sigma-Aldrich, Missouri, United States) for actin staining. After final washing steps in PBS, cells were mounted with fluoroshield containing DAPI for nuclei staining (Sigma-Aldrich, Germany).

Samples were imaged with a Leica TCS SP8 LSCM (Leica Microsystems CMS GmbH, Mannheim, Germany) using the acquisition software LAS-X (Vers. 3.5.0.18371, Leica Microsystems CMS GmbH, Mannheim, Germany) for high-resolution images. 20x oil immersion objective was selected to record an overview of Cx43 stainings, and 63x oil immersion objective was used for detailed imaging of SERCA, NCX1, RyR2, and Ca_v_1.2 expression. Laser excitation at 405nm, 488nm, 552nm, and 638nm was used in sequential scans. Emission spectra were chosen *via* tunable filters, and emission detection was achieved by photomultiplier tubes (PMT) and hybrid detectors (HyD). Image processing and Cx43 sarcolemmal expression analysis were done using ImageJ/Fiji as described in (Sottas et al., 2018). Due to the strong light absorbance of PDMS-coated surfaces, weak specific fluorescence signals were denoised using the ImageJ plugin PureDenoise developed by Florian Luisier (EPFL, Switzerland).

### Data Analysis and Statistics

For statistical data analysis and graph design, OriginPro^®^ software (OriginLab Corporation, Northampton, MA, United States) was used. Images were processed in ImageJ. Data are presented as mean±standard error of the mean (SEM) with n equaling the number of individually analyzed cells. Experiments were repeated in five rounds with iPSC-CMs defrosted from five individual vials at different time points (5 vials à 1 Mio cells, acquired from Ncardia). Depending on the data set, statistical significance was determined by Student’s *t*-tests or one-way ANOVA followed by multiple comparison tests, indicated by ^*^ for *p*<0.05.

## Results

### Impact of Different Growth Surface Stiffnesses on iPSC-CMs Contractility

For live-imaging and functional analysis, cells are usually seeded on rigid glass surfaces, which present an unphysiologically high stiffness of 25GPa. In order to investigate the impact of different physiological substrate stiffnesses on the maturation potential of iPSC-CMs, cells were seeded at high density on glass and for comparison on PDMS culture surfaces with stiffnesses of 28kPa, 15kPa, and 1.5kPa, respectively, corresponding to the environment of neonatal and adult cardiomyocytes. All surfaces were coated with laminin and fibronectin for cell adhesion. Different growth materials did not have any impact on cell adhesion or cell survival during the culture time of up to 4weeks (data not shown). But a simple examination of cell contractile behavior revealed that compared to glass, the contractile activity of iPSC-CMs appeared stronger and temporally better synchronized across the cell monolayer (Supplementary Video S1–S4) indicating increased contractility and intercellular connectivity. To quantify these observations, we investigated EC-coupling and intercellular communication in these cells.

### Effects of Different Substrate Stiffnesses on Ca^2+^ Handling and Contractility in iPSC-CMs

**Figure 1:**
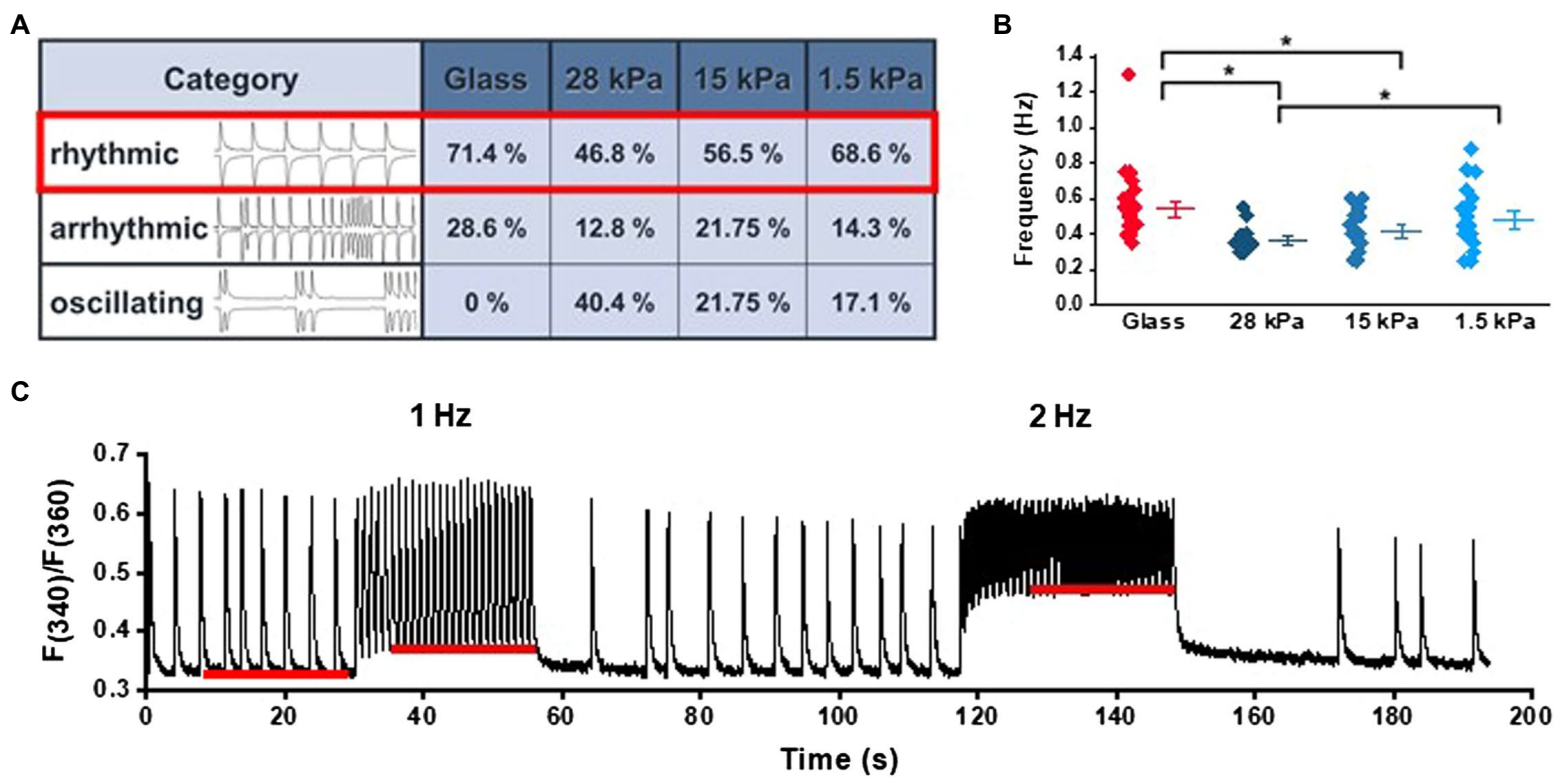
Ca^2+^ signaling in iPSC-CMs grown on substrates with different stiffnesses during spontaneous activity and electrical pacing. **(A)** Categorization of different phenotypes of spontaneous activity ranging from rhythmic, arrhythmic to oscillating activity patterns in cells grown on surfaces with different stiffnesses (*n*=42 on glass, *n*=47 on 28kPa-PDMS, *n*=44 on 15kPa-PDMS, and *n*=35 on 1.5kPa-PDMS). **(B)** Quantification of the spontaneous beating frequency of rhythmic cells (glass: *n*=30; 28kPa-PDMS: *n*=21; 15kPa-PDMS: *n*=25; and 1.5kPa-PDMS: *n*=24). **(C)** Sample trace of Ca^2+^ transients recorded during spontaneous activity and during episodes of electrical pacing at 1Hz or 2Hz. The red lines illustrate frequency-dependent changes of diastolic Ca^2+^ levels.

To evaluate Ca^2+^ handling and contractile activity in iPSC-CMs, spontaneous Ca^2+^ transients and contraction dynamics were measured in cells seeded on PDMS-coated or glass-bottom dishes. Cells were loaded with the fluorescent Ca^2+^-sensitive dye fura-2AM (1.5μm) to record Ca^2+^ transients, and cell shortening was optically measured *via* edge detection. Due to their immaturity, iPSC-CMs showed high variability in their spontaneous activity pattern. For evaluation of comparable cells, we categorized iPSC-CMs into three different groups of rhythmic, arrhythmic, and oscillating cells (Figure 1A). In order to avoid high variability in the experiments, only rhythmically beating cells were included for further analysis. Figure 1A demonstrates that the ratio of rhythmically beating cells, as assessed from Ca^2+^ transients’ measurements, was higher on glass and on 1.5kPa-PDMS compared to 28kPa-PDMS and 15kPa-PDMS. In contrast to iPSC-CMs seeded on glass, oscillating spontaneous activity was only observed in cells grown on PDMS-coated dishes. According to the distribution of spontaneous activity patterns, rhythmic iPSC-CMs revealed higher spontaneous beating frequencies when grown on glass or 1.5kPa-PDMS compared to 28kPa-PDMS and 15kPa-PDMS (Figure 1B). In Figure 1C, representative recordings of Ca^2+^ transients illustrate the response to different pacing frequencies, as well as the frequency-dependent changes of diastolic Ca^2+^ levels at 1 and 2Hz. As shown in Supplementary Table S1, the statistical evaluation revealed similar diastolic Ca^2+^ levels in iPSC-CMs during spontaneous beating activity on all tested growth surfaces. Pacing at 1 and 2Hz slightly enhanced cytosolic basal Ca^2+^ levels on all tested growth surfaces, indicating immature Ca^2+^ handling processes especially at higher stimulation frequencies. Diastolic Ca^2+^ levels revealed no significant differences except for iPSC-CMs on 28kPa-PDMS compared to glass during pacing at 2Hz.

### Characterization of CICR in iPSC-CMs Grown on Substrates With Different Stiffnesses

**Figure 2:**
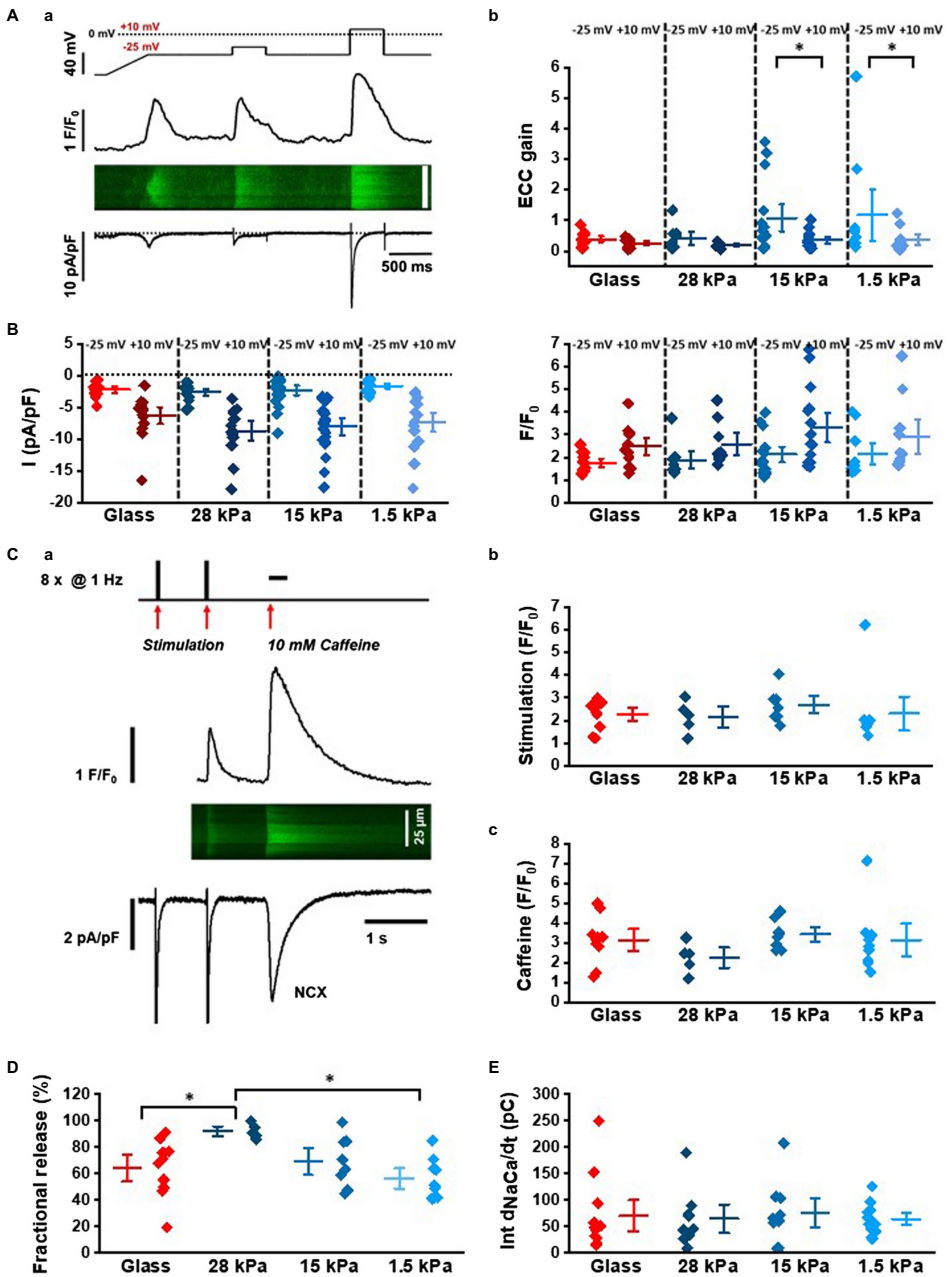
Characterization of Ca^2+^-induced Ca^2+^ release in patch-clamped iPSC-CMs grown on surfaces with different matrix stiffnesses. **(Aa)** Stimulation protocol and representative recordings of *I*_CaL_, Ca^2+^ transients in the line-scan mode and line profile of the line-scan. **(Ab)** Analysis of EC-coupling gain at −25mv and+10mv calculated from peak *I*_CaL_ and Ca^2+^ transient amplitudes. These data sets were statistically compared by two-way ANOVA with Holm-Sidak’s post-hoc pairwise multiple comparisons *vs.* control. **(B)** Peak *I*_CaL_ (left) and Ca^2+^ transient amplitudes (right) at −25mv and+10mv in cells seeded on different stiffnesses. **(C)** Assessment of NCX activity and fractional SR release with caffeine: **(Ca)** Representative line-scan image, line profile of Ca^2+^ transients, and membrane current evoked by 1Hz-steady-state activation and caffeine-induced Ca^2+^ release (10mM caffeine). **(Cb)** Ca^2+^ transient amplitudes during steady-state activation (*n*=9–15 experiments). **(Cc)** Peak amplitudes of caffeine-induced Ca^2+^ transients (*n*=5–10 experiments). **(D)** Fractional release calculated from the ratio of peak Ca^2+^ transient amplitudes during electrical pacing and caffeine-induced Ca^2+^ release. **(E)** Integrated NCX membrane currents measured during caffeine exposition in iPSC-CMs grown on substrates with different stiffnesses.

To investigate CICR in more detail, we measured membrane Ca^2+^ currents (*I*_CaL_) using the whole-cell patch-clamp technique and recorded simultaneously intracellular Ca^2+^ transients by confocal imaging in the line-scan mode. As demonstrated in Figure 2A, for electrical stimulation *via* patch-clamp, a two-step protocol was applied to measure *I*_CaL_ at negative potentials and at maximal current activation. In parallel, changes in cytosolic Ca^2+^ levels were recorded by confocal imaging of fluo-3 included in the patch pipette solution. Starting first from a holding potential of −80mv, a 500ms voltage-ramp to −40mv was applied to activate and immediately inactivate the fast voltage-dependent Na^+^ current. After 800ms at −40mv, the first test step was applied. E_m_ was set to −25mv to activate *I*_CaL_ and CICR at low amplitude. The second test step to +10mv fully activated *I*_CaL_ and served as control measurement for *I*_CaL_ and CICR. Changes in fluorescence intensity were plotted over time to generate line profiles from the line-scan image, as depicted in Figure 2Aa. Analysis of peak *I*_CaL_ and Ca^2+^ transients at +10mv showed similar amplitudes in iPSC-CMs grown on substrates with different stiffnesses (Figure 2B). Data are summarized in Supplementary Table S2. The EC-coupling gain was calculated from the ratio of the peak Ca^2+^ transient amplitude and peak *I*_CaL_ at −25mv and at +10mv. The data showed an increased EC-coupling gain on soft surfaces (Figure 2Ab), which is also reflected by the increase in variability of the individual gain values at −25mv compared to +10mv (coefficient of variation C_var_: 0.49 for glass, 0.61 for 28kPa-PDMS, 0.9 for 15kPa-PDMS, and 1.12 for 1.5kPa-PDMS). The EC-coupling gain at −25mv was significantly increased in comparison with +10mv when stiffness was 15kPa (*p*=0.0445) or 1.5kPa (*p*=0.0453; Supplementary Table S2).

In order to investigate SR Ca^2+^ load, the SR Ca^2+^ content was assessed by caffeine-mediated Ca^2+^ release (10mM) in iPSC-CMs grown on surfaces with different stiffness. Figure 2Ca illustrates the experimental protocol: After steady-state stimulation at 1Hz in patch-clamped cells, caffeine was applied. NCX currents and Ca^2+^ transients were recorded simultaneously. Peak amplitudes of steady-state Ca^2+^ transients and caffeine-induced Ca^2+^ transients are summarized in Figures 2Cb,2Cc, respectively, and Supplementary Table S3 demonstrating a similar SR Ca^2+^ load (Figure 2Cc) and fractional release (Figure 2D) at the different conditions. NCX currents were measured during Ca^2+^ release evoked by prolonged caffeine application. By integration of inward currents, total charge movement across the sarcolemma was measured indicative of global NCX activity. Statistical analysis revealed similar NCX activity in iPSC-CMs grown on substrates with different stiffnesses (Figure 2E; Supplementary Table S3).

### Structural Remodeling of Ca_v_1.2 and RyR2 Expression in iPSC-CMs Grown on Soft Surfaces

**Figure 3:**
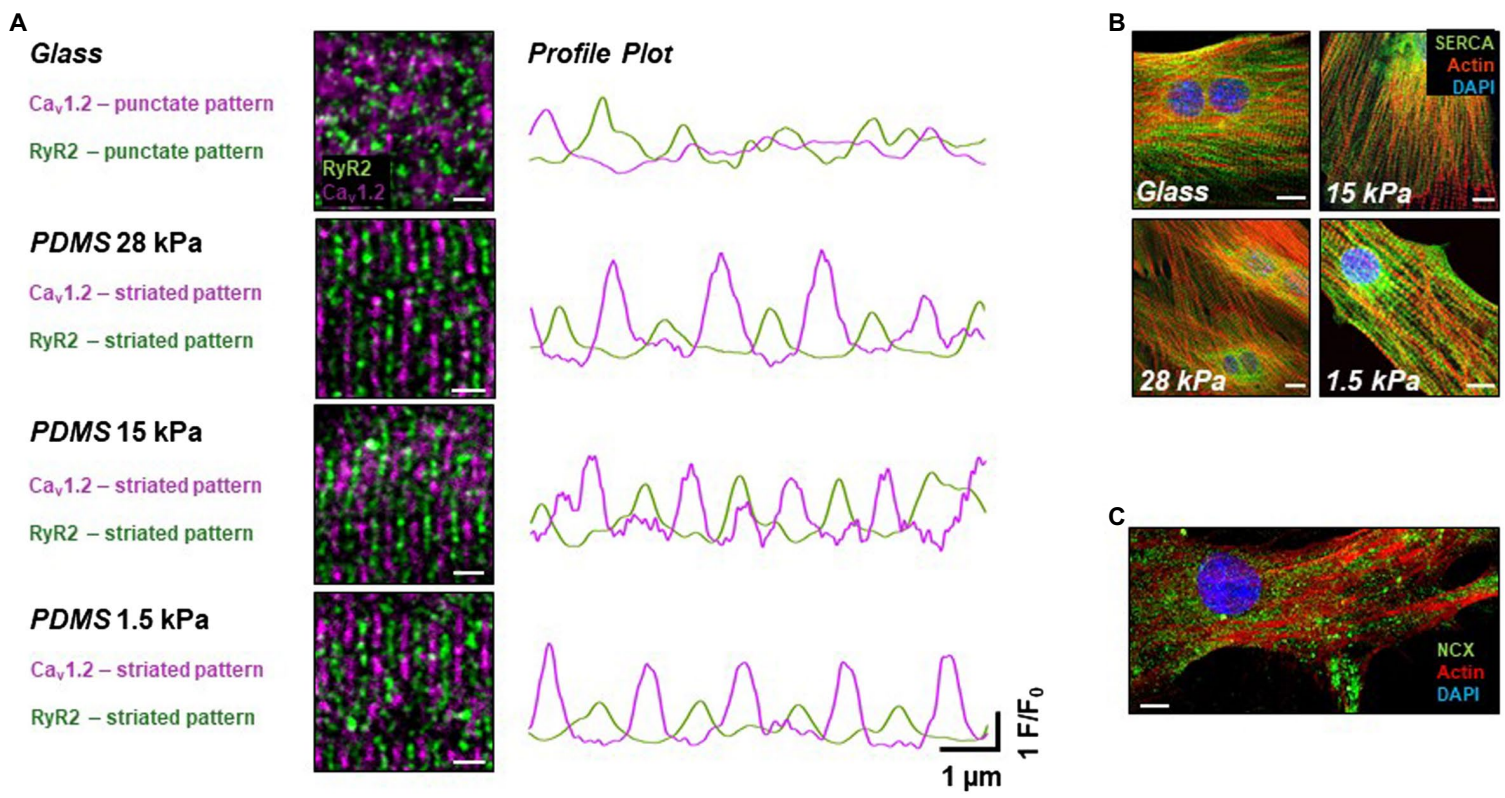
Expression pattern of Ca^2+^ handling proteins in iPSC-CMs grown on different soft surfaces. **(A)** Immunostainings of L-type Ca^2+^ channel Ca_v_1.2 (purple) and RyR2 [green, scale bar (SB)=2μm] and intensity profiles drawn from regions of interest along the vertical axis indicating parallel alignment of both ion channel expression patterns relative to each other by alternating peaks in iPSC-CMs plated on soft surfaces compared to glass. **(B)** Expression pattern of SERCA (green), actin (red), and nucleus (blue) revealing similar distribution of the SR network in iPSC-CMs grown on all tested surfaces (SB=10μm). **(C)** Representative image of NCX stainings (green) together with actin (red) and nucleus (blue) showing a dotted distribution all over the sarcolemma (SB=10μm).

We further investigated different Ca^2+^ handling proteins on a structural level. As recently published by our group, functional maturation, such as improved cytosolic Ca^2+^ handling, during EC-coupling can be triggered by structural remodeling in iPSC-CMs (Silbernagel et al., 2020). Here, we considered subcellular structural reorganization as a possible cause for the apparent changes in the EC-coupling gain in iPSC-CMs grown on soft surfaces. To examine the expression pattern of proteins relevant for CICR, the LTCC α-subunit Ca_v_1.2, RyR2, SERCA, and NCX were stained in immunocytochemical assays in iPSC-CMs grown on substrates of different stiffnesses for 20days (Figure 3). In total, 12 dishes of 4 different stiffnesses from 3 different time points were stained and imaged. Interestingly, while the expression pattern of Ca_v_1.2 exhibited a dotted pattern in cells grown on glass, a pronounced striation pattern of Ca_v_1.2 expression formed on all soft growth surfaces after 20days in culture (Figure 3A). Structural remodeling of the investigated Ca^2+^ channels and Ca^2+^ handling proteins may happen early during culture on soft PDMS-coated culture surfaces, as already on day 10, cells showed the same expression pattern of the investigated proteins as on day 20. Moreover, the images demonstrate a high organization level of RyR2 expression, again in a striated manner, in cells grown on soft surfaces indicating a well-developed SR network. On the contrary, in iPSC-CMs grown on glass, RyR2 staining revealed only a punctate expression pattern, pointing toward a rather immature distribution of RyR2 and SR organization within these cells. Overview images of Ca_v_1.2 and RyR2 expression are shown in Supplementary Figure S1. Merging the expression pattern of Ca_v_1.2 and RyR2 revealed an alternating parallel alignment of both Ca^2+^ channels in iPSC-CMs grown on soft surfaces suggesting close localization to each other. As shown in Figure 3A on the right side, line profiles of LTCC and RyR2 expression confirmed the high degree of parallel alignment as indicated by the alternating intensity peaks of fluorescence derived from the ion channels’ expression pattern. Notably, this structural proximity is essential for efficient functional coupling of LTCC and RyR2 during CICR, which may lead to improvement of the EC-coupling mechanism – indicative of a beginning functional maturation. Furthermore, the expression pattern of SERCA was investigated in iPSC-CMs grown on substrates with different stiffnesses for 20–25days. Images are summarized in Figure 3B and demonstrate a dense network (in green) reaching toward the cell periphery lining the actin filaments of the myofibrils as well as strong perinuclear expression. Detailed images are shown in Supplementary Figure S2. This expression pattern of SERCA demonstrates a well-organized subcellular arrangement of the SR. However, no significant difference of SERCA expression depending on the substrate stiffnesses was observed in iPSC-CMs grown on different surfaces. Figure 3C and Supplementary Figure S3 show a representative picture of the sarcolemmal distribution of NCX in iPSC-CMs examined by immunocytochemistry. In total, 12 dishes of 4 different stiffnesses from 3 different time points were stained and imaged. The expression pattern of NCX revealed a dotted distribution over the entire sarcolemma of iPSC-CMs, which was similar in cells grown on different surfaces. Occasionally, small areas reveal stretches of more continuous NCX signals (Supplementary Figure S3).

### Influence of Environmental Stiffness on Ca^2+^ Transients and Contractions

**Figure 4:**
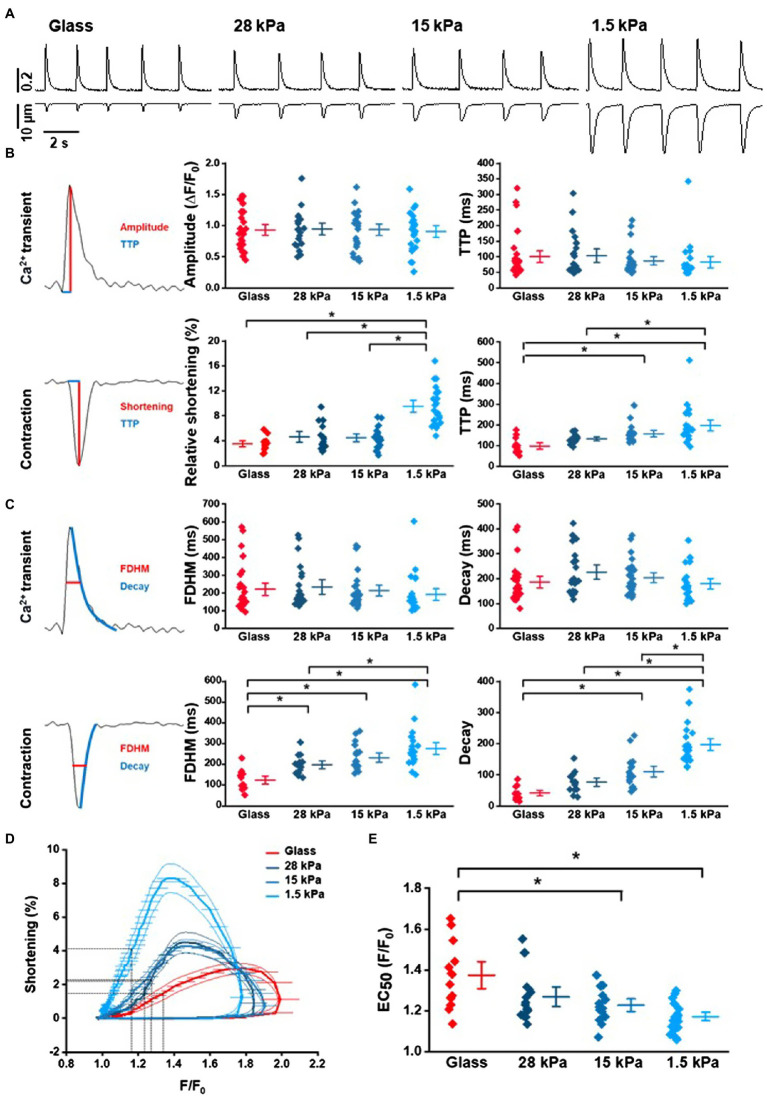
Ca^2+^ transient and contraction analysis in iPSC-CMs depending on of growth surface. **(A)** Representative traces of spontaneous Ca^2+^ transients and cell shortening of iPSC-CMs grown on glass or PDMS with 28kPa, 15kPa, or 1.5kPa. **(B)** Analysis of peak amplitudes and time-to-peak (TTP) of Ca^2+^ transients and relative cell shortening in rhythmically beating cells (*n*=13–23 measurements). Statistical evaluation revealed significantly larger shortening amplitudes in cells grown on 1.5kPa compared to any other surface stiffness, and slower TTP of contractions on 1.5kPa compared to 28kPa or glass and on 15kPa compared to glass (one-way ANOVA, *p*<0.05). **(C)** Analysis of FDHM and decay kinetics of Ca^2+^ transients and contractions on glass compared to soft surfaces. **(D)** Analysis of Ca^2+^ sensitivity in Ca^2+^ loops revealing increased myofilament Ca^2+^ sensitivity in iPSC-CMs grown on 1.5kPa and 15kPa compared to glass. Data are taken from the traces of Ca^2+^ transients (x-axis) and cell shortening (y-axis). **(E)** Statistical analysis of the EC_50_ values during the relaxation phase from **(D)**. Statistically significant differences are indicated by ^*^ for *p*<0.05.

In order to further investigate cytosolic Ca^2+^ handling and Ca^2+^-induced contraction properties in iPSC-CMs on growth surfaces with different stiffnesses, Ca^2+^ transients and cell shortening were measured and analyzed simultaneously. Representative Ca^2+^ and contraction traces of rhythmically beating iPSC-CMs are depicted in Figure 4A. While Ca^2+^ transients exhibited similar characteristics on different surfaces (peak amplitude, TTP, FDHM, and decay time), relative shortening amplitude was significantly larger in iPSC-CMs grown on 1.5kPa-PDMS compared to higher stiffnesses of glass, 28kPa-PDMS or 15kPa-PDMS, indicating significantly enhanced contractility (Figure 4B). All data from these experiments are summarized in Supplementary Table S4. Moreover, TTP of contractions was significantly slower in cells grown on 1.5kPa-PDMS surfaces than on 28kPa-PDMS and glass and on 15kPa-PDMS compared to glass (Figure 4B). Additionally, analysis of contractions revealed significantly and increasingly longer durations (expressed as FDHM) in iPSC-CMs plated on soft surfaces than on glass (Figure 4C) suggesting slower relaxation on soft surfaces as reflected by significantly slower decay times in cells on 1.5kPa-PDMS compared to 15kPa-PDMS, 28kPa-PDMS, and glass. To investigate whether growth surface stiffness influences Ca^2+^ removal properties after release, Ca^2+^ transient decay dynamics were analyzed. SERCA function was assessed by fitting the decay of Ca^2+^ transients with a monoexponential function to obtain the time constant of Ca^2+^ removal (Figure 4C). While Ca^2+^ removal dynamics were not different, cell relaxation expressed as the decay of shortening was significantly prolonged on soft surfaces.

To investigate the underlying cause of the altered contractile dynamics of iPSC-CMs on soft substrates, cell shortening and relaxation were plotted as a function of the intracellular Ca^2+^ concentration. The resulting phase-plane diagrams are depicted in Figure 4D. Presented Ca^2+^-contraction loops revealed a leftward shift of the relaxation phase in iPSC-CMs grown on soft surfaces indicating an increase in the Ca^2+^ sensitivity of the myofilaments. Statistical analysis of the Ca^2+^ concentration at half-maximal relaxation (EC_50_) on substrates with different stiffnesses revealed significantly higher myofilament Ca^2+^ sensitivity in iPSC-CMs seeded on 1.5kPa-PDMS and 15kPa-PDMS compared to glass (Figure 4E). All data from these experiments are summarized in Supplementary Table S4.

### Influence of Different Substrate Stiffnesses on Cx43 Expression Pattern

**Figure 5:**
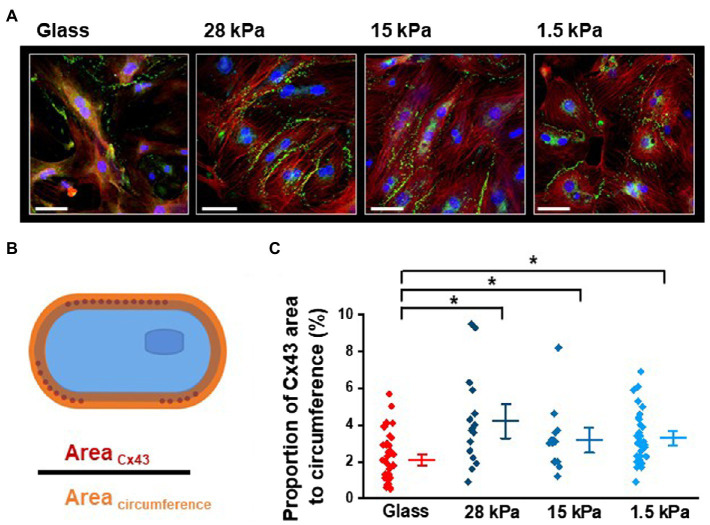
Influence of different growth surface stiffnesses on Cx43 expression in iPSC-CMs. **(A)** Representative images showing Cx43 expression in green and nuclear signal in blue. Cx43 is characteristically located at the cell borders in iPSC-CMs, but is also expressed in the perinuclear regions (SB=50μm). **(B)** Illustration of the quantitative analysis of Cx43-occupied area of the cell membrane in relation to the area of the entire sarcolemma. **(C)** Statistical evaluation of the proportion of Cx43-occupied area to the circumference revealing enhanced sarcolemmal expression of Cx43 in iPSC-CMs grown on soft surfaces compared to glass. Glass: *n*=40; 28kPa-PDMS: *n*=16; 15kPa-PDMS: *n*=14; and 1.5kPa-PDMS: *n*=21.

In the next set of experiments, we focused on the second major observation of improved and synchronous beating activity in iPSC-CMs grown on soft surfaces compared to glass. To investigate the expression pattern of Cx43 in iPSC-CMs, we performed immunostainings with cells grown on substrates with different stiffnesses for 10–13days. In total, 12 dishes of 4 different stiffnesses were processed at 3 different time points. Figure 5A shows representative images of the typical localization of Cx43 (in green) at cell borders and in perinuclear areas in iPSC-CMs plated on glass, 28kPa-PDMS, 15kPa-PDMS, and 1.5kPa-PDMS. To quantify only the Cx43 expression that is relevant to form gap junctions for intercellular coupling, we calculated the proportion of the Cx43-occupied area of the cell membrane to the cell’s entire circumference, as illustrated in Figure 5B. Statistical evaluation revealed a significantly higher ratio of Cx43 expression at the cell membrane in iPSC-CMs grown on soft surfaces comprising 28kPa-PDMS, 15kPa-PDMS, and 1.5kPa-PDMS compared to glass (Figure 5C). To support this quantification, we further examined intercellular coupling in iPSC-CMs grown on substrates with different stiffnesses at the functional level.

### Enhanced Intercellular Coupling on Soft Surfaces

**Figure 6:**
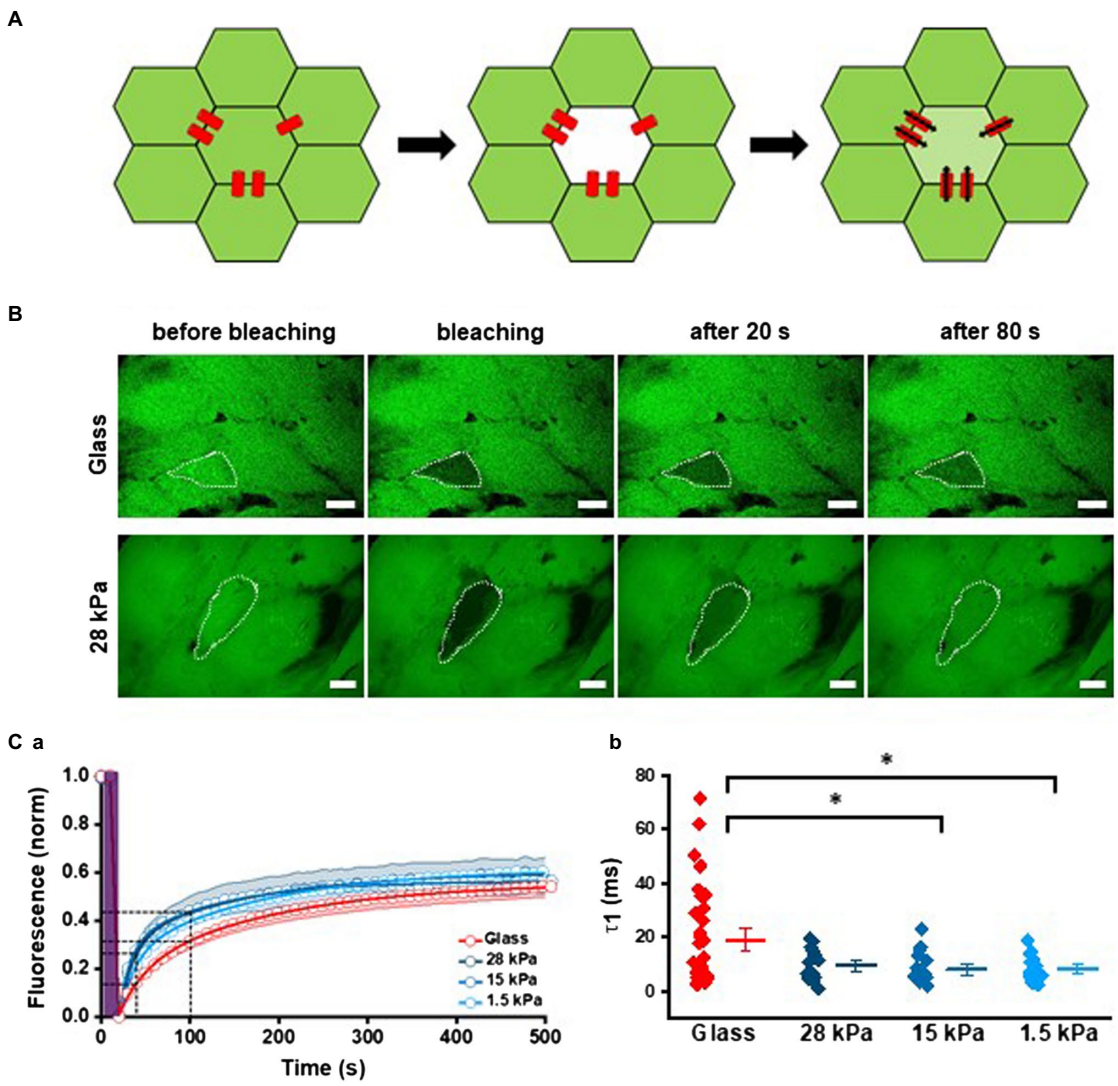
Enhanced intercellular coupling on soft surfaces. **(A)** Schematic illustration of FRAP experiments. *Left:* the cell layer is stained with calcein (shown in green). *Middle:* one cell within a cell cluster is bleached by high laser power (5s). *Right:* after bleaching recovery of fluorescence by calcein diffusion through gap junctions from neighbored cells is monitored over time. **(B)** Representative FRAP experiments with iPSC-CMs grown on glass and 28kPa. The target cell is marked by a white dotted line. The first image corresponds to *t*=0s before bleaching, and the second image was taken at *t* =20s immediately after bleaching. At *t*=40s and 100s, the third and fourth images were taken. **(Ca)** Average time courses of FRAP for each stiffness were plotted and fitted with a biexponential function. FRAP was significantly faster in iPSC-CMs grown on all PDMS-coated surfaces compared to glass. **(Cb)** Statistical evaluation of tau1 (τ1). For cells grown on glass *n*=35; for 28kPa-PDMS *n*=15; for 15kPa-PDMS *n*=17; and for 1.5kPa-PDMS *n*=18.

For functional evaluation of gap junctions, we analyzed FRAP using the gap junction permeant fluorescent dye calcein-AM (0.5mM). After removal of the acetoxy-methylester group of calcein by cytosolic esterases, the dye was no longer able to diffuse across the lipid bilayer. Consequently, gap junctions presented the only possibility for calcein to leave intact cells. Calcein was bleached in one cell of a cell group, and fluorescence recovery of calcein diffusing in from neighboring cells was measured over time revealing the diffusion dynamics between iPSC-CMs (Figure 6A). Representative images of treated iPSC-CMs grown on glass and 28kPa-PDMS before, during, and after bleaching are depicted in Figure 6B. In contrast to cell bleaching on the glass surface, which showed only 14% of fluorescent recovery at 20s and 32% at 80s after bleaching, fluorescence recovery of a bleached cell grown on 28kPa-PDMS amounted to 29% and 44% within the same time frame. For analysis of fluorescence recovery, fluorescence intensity was plotted over time and fitted with a biexponential function to calculate the diffusion rate constants (Figure 6Ca). Figure 6Cb summarizes the first time constant (τ1) of the recovery time course revealing a steeper slope within the first 100s of FRAP in iPSC-CMs grown on all soft surfaces compared to glass. Statistical evaluation of τ1 confirmed significantly faster diffusion rates in cells seeded on 28kPa-PDMS, 15kPa-PDMS, and 1.5kPa-PDMS compared to glass. In addition, the variability of τ1, assessed by C_var.,_ is significantly reduced in cells grown on soft surfaces compared to glass (C_var_: Glass: 0.92, 28kPa-PDMS: 0.63, 15kPa-PDMS: 0.73, and 1.5kPa-PDMS: 0.54). These results demonstrate significantly enhanced diffusion dynamics of calcein and therefore an increased presence of functional gap junctions, which allowed faster FRAP in iPSC-CMs grown on soft surfaces.

## Discussion

For the development of an adult heart with its unique electrophysiological and contractile properties, numerous specific growth conditions are required. Electrical stimulation by pacemaker cells, humoral regulation, and the balance between extrinsic and intrinsic mechanical load are just some of the essential factors that influence structural and functional maturation of cardiomyocytes during the early stages of development (Zhu et al., 2014). In this context, another important environmental cue is mediated by the composition of the ECM defining the stiffness surrounding each cell and therefore the passive resistance that cardiomyocytes must pull against during contraction. Modification of this environmental stiffness during cardiac development may have a significant impact on the transition of early to adult cardiomyocytes (Ward and Iskratsch, 2020). Moreover, pathophysiological ventricular remodeling upon cardiac fibrosis and heart disease suggests activation of different signaling pathways in response to ECM restructuring (Sit et al., 2019). This process is also called mechano-chemotransduction (MCT; Chen-Izu and Izu, 2017). Although not fully understood yet, some molecular players and signaling pathways have already been identified in adult and developing cardiomyocytes (Gaetani et al., 2020; Izu et al., 2020; Ward and Iskratsch, 2020).

Considering cardiomyocytes derived from pluripotent stem cells, investigations of MCT may reveal new targets for improving their functional properties to enhance their potential to be used for cardiac cell therapy. In other words, a detailed analysis of the influence of specific environmental cues on elementary features of iPSC-CMs may help to mature these cells *in vitro*. The goal of this study was therefore to examine the impact of the environmental stiffness on cardiomyocyte function at the level of EC-coupling and intercellular communication in iPSC-CMs and to find out whether an environment with physiological stiffness may provide a new possibility to enhance mature properties of iPSC-CMs.

### Enhanced Contractility in iPSC-CMs Grown on Soft Surfaces

EC-coupling, the fundamental mechanism linking electrical excitation to contractile activity and force production, is strictly controlled by CICR. On a structural level, efficient EC-coupling requires close interaction of LTCCs and RyR2s, which is usually provided by the regular formation of dyads, where t-tubular and SR membranes come close together and enable spatiotemporally synchronized CICR. Deviations from the optimized microarchitecture of adult cardiomyocytes are seen in immature prenatal and iPSC-CMs or in diseased cardiomyocytes, where such a missing membrane organization leads to an enhanced appearance of so-called orphaned RyR2s (Lee et al., 2011; Rao et al., 2013). Examination of the EC-coupling gain not only helps to identify weaknesses in this mechanism but also allows to detect improvements in Ca^2+^ handling in response to a specific treatment. iPSC-CMs plated on soft surfaces of 15 or 1.5kPa-PDMS showed greater Ca^2+^ release upon triggering influx current at −25mv, indicative of better coupling between LTCCs and RyR2s. Considering possible explanations for this enhanced EC-coupling gain, changes in the expression pattern of LTCCs and RyR2s, i.e., a switch from punctuate to regular striation pattern, may explain this finding very well and indicate structural remodeling toward maturation. In this new arrangement, the number of non-coupled RyR2s is reduced, while the increased colocalization of LTCCs and RyR2s favors higher EC-coupling efficiency in iPSC-CMs. Another possibility for the enhanced EC-coupling gain might be an increase in the Ca^2+^ sensitivity of RyR2s due to posttranslational modifications. In addition to reactive oxygen and nitrogen species, Ca^2+^-dependent RyR2 activation is physiologically regulated by phosphorylation *via* protein kinase A (PKA) and Ca^2+^/calmodulin-dependent kinase II (CaMKII; Niggli et al., 2013). Interestingly, increased RyR2 sensitivity mediated by the neuronal isoform of the nitric oxide synthase (nNOS) and CaMKII was shown to be induced by multiaxial mechanical stress during cardiomyocyte contraction (Jian et al., 2014; Mosqueira et al., 2021). As cellular afterload response results in modulation of RyR2 activity in adult cardiomyocytes, substrates with different stiffnesses may influence signaling pathways leading to altered Ca^2+^ sensitivity of RyR2s in iPSC-CMs as well. Moreover, NCX stainings revealed small stretches of sarcolemmal invaginations from the surface into the cell body. It is only by speculation that one can assume here the beginning of t-tubular-like structures, but this cannot be excluded. Enhanced t-tubular structures in remodeled iPSC-CMs may even better explain the increase in the EC-coupling gain. Further experiments will be needed to investigate the initiation of t-tubules by environmental cues. Therefore, our findings suggest that cardiac-like environmental stiffness induces enhanced EC-coupling gain at the level of CICR by activation of intracellular signaling cascades inducing structural remodeling of LTCC and RyR2 and modifying RyR2 function.

Moreover, a deeper investigation of EC-coupling revealed altered contraction dynamics with increased shortening in iPSC-CMs plated on substrates with softer stiffnesses, overall leading to larger contractions. As one good reason for impaired contractility on rigid surfaces, we first assumed that high stiffness may prevent adherent iPSC-CMs from contracting to their full extent. In contrast, on flexible surfaces, the contractile cells may pull against the soft material, leading to higher contraction amplitudes and longer contraction duration. However, there was no linear relationship between stiffness and change in contractility across the large range of tested surface stiffnesses indicating additional mechanisms responsible for functional changes apart from passive inhibition. Supporting this evidence, van Deel et al. distinguished between passive inhibitory effects and active changes induced by environmental stiffness by acutely detaching adult cardiomyocytes from their growth surface just before contractility was measured. They demonstrated that the functional adaptations of the cardiomyocytes were independent of the direct passive effect of matrix rigidity on cell function (van Deel et al., 2017). This study supports our idea that changes in environmental stiffness induce intrinsic cellular effects that alter contractile dynamics in iPSC-CMs. Although the exact underlying mechanism remains to be determined, changes in Ca^2+^ handling or modifications of myofilament proteins were discussed.

Considering active matrix-induced alterations, we wanted to find out whether MCT may influence signaling pathways leading to improved EC-coupling with increased contractility. As a possible explanation for enhanced contractile dynamics in iPSC-CMs grown on soft surfaces, differences in Ca^2+^ handling may be considered, since an increase in the EC-coupling gain means that at the same electrical stimulus (*via I*_CaL_), more Ca^2+^ is being released from the SR and therefore available for myofilament activation. In addition, the contractile response to different surfaces in adult cardiomyocytes may also depend on adaptions in myofilaments, an idea that also seems to apply to iPSC-CMs (Galie et al., 2013). Our data support previous evidence, where decreased sarcomere and myofibril activity due to intracellular over-tension were shown in iPSC-CMs grown on 35kPa hydrogels leading to 90% less mechanical output compared to iPSC-CMs seeded on softer surfaces of 10kPa or 6kPa hydrogels (Ribeiro et al., 2015). Nevertheless, myofilament buckling during relaxation in iPSC-CMs plated on 6kPa indicated the need for some intracellular tension to maintain correct myofibril alignment (Ribeiro et al., 2015). These results correspond to preferred ranges of matrix elasticity for optimal contractile work in embryonic and neonatal cardiomyocytes (Engler et al., 2008; Bhana et al., 2010). Therefore, an intermediate stiffness comparable to native myocardium may optimally maturate myofilament organization and function. Moreover, a more robust expression of cardiac troponin I (cTnI) was found in neonatal and iPSC-CMs seeded on soft surfaces (Bhana et al., 2010; Ribeiro et al., 2015; Herron et al., 2016). Thus, changes in myofilament Ca^2+^ sensitivity present a reasonable cause for altered contraction dynamics in iPSC-CMs seeded on substrates with lower stiffness. In the phase-plane diagram, cell shortening and relaxation were plotted as a function of the intracellular Ca^2+^ concentration during a twitch. Our data revealed a pronounced leftward shift of the relaxation phase in iPSC-CMs grown on soft surfaces indicating higher myofilament Ca^2+^ sensitivity. In principle, changes in myofilament Ca^2+^ sensitivity are evoked by modified on- and off-rates of myofilament activation, which are mainly, but not exclusively caused by altered association and dissociation rates of Ca^2+^ to cardiac troponin C (cTnC; Chung et al., 2016). Considering matrix-induced changes in gene expression and long-term structural adaptations, enhanced myofibril organization may contribute to increased myofilament Ca^2+^ sensitivity in iPSC-CMs grown on soft surfaces (Young et al., 2014).

Taken together, investigation of EC-coupling and contraction demonstrated a significant influence of growth surface properties on structural maturation at the level of CICR and myofilament activation. Therefore, providing an environment with natural stiffness improves the contractile potential of iPSC-CMs toward more mature and physiological function.

### Increased Intercellular Coupling in iPSC-CMs Grown on Soft Surfaces Due to Improved Cx43 Expression

For functional integration of engineered myocardium in diseased adult hearts, adequate propagation of excitation across the cardiac tissue and graft is essential for coordinated impulse propagation. Slow and irregular electrical signal transmission in embryonic (ESC-) and iPSC-CMs caused by weak intercellular coupling poses a high risk for the development of a conduction barrier and arising arrhythmias (Shiba et al., 2012; Kucera et al., 2015; Sottas et al., 2018). In our experimental approach, we tested the hypothesis that growth surfaces with different stiffnesses affect cell-cell communication in iPSC-CMs by influencing the expression pattern of Cx43. Our evaluation of Cx43 immunostainings showed an increased expression of Cx43 at the sarcolemma indicating enhanced gap junction formation, which was functionally confirmed by FRAP analysis: iPSC-CMs grown on soft surfaces revealed faster diffusion rates. Assuming substrate stiffness as the triggering factor, several steps in the Cx43 lifecycle can be considered as possible target points: Critical to intercellular communication are transcription factors influencing gene expression, endoplasmic reticulum and Golgi assembly and transport, forward trafficking to the sarcolemma, organization within the gap junction plaque, and retrograde transport for degradation (Zhang and Shaw, 2014). Most of these processes are regulated by posttranslational modifications, and as Cx43 has a high turnover rate, especially the process of trafficking may also play an important role during remodeling. Recently, Herron et al. suggested enhanced α5β1-integrin receptor activation as a possible reason for increased intercellular coupling in iPSC-CMs grown on soft surfaces (Herron et al., 2016). Indeed, integrins are considered the main receptors associated with sensing mechanical signals and changes in load (Ward and Iskratsch, 2020). In the developing heart, for example, repression of integrin α5 during early stages results in reduced cardiomyocyte differentiation and impaired contractility (Neiman et al., 2019). Therefore, further investigations of integrin receptor expression depending on the environmental stiffness may reveal novel approaches to promote maturation in iPSC-CMs.

In conclusion, here we showed non-linear cellular responses to substrates with different stiffnesses at a macroscale level in iPSC-CMs indicating the existence of MCT. We demonstrated that soft growth surfaces trigger structural maturation at the level of CICR and promote Ca^2+^ handling properties leading to enhanced EC-coupling gain and contractility in iPSC-CMs. Moreover, this study provides the first experimental evidence of an increased presence of functional gap junctions, which lead to better intercellular coupling with stronger synchronization of the electro-mechanical activity in iPSC-CMs grown on soft surfaces. By exhibiting relevant targets of MCT in iPSC-CMs, our results may trigger further investigation of mechanical signaling pathways leading not only to new maturation strategies of iPSC-CMs, but also to the identification of new targets for the treatment of cardiac diseases with changed ECM stiffness.

## Data Availability Statement

The original contributions presented in the study are included in the article/Supplementary Material, further inquiries can be directed to the corresponding author.

## Author Contributions

NU conceived the study. AK and NU designed the experiments, conducted the experiments, analyzed and interpreted the data, generated the figures and wrote the manuscript. MM and MH critically reviewed and edited the manuscript. All authors have reviewed the data and approved the final manuscript.

## Conflict of Interest

The authors declare that the research was conducted in the absence of any commercial or financial relationships that could be construed as a potential conflict of interest.

## Publisher’s Note

All claims expressed in this article are solely those of the authors and do not necessarily represent those of their affiliated organizations, or those of the publisher, the editors and the reviewers. Any product that may be evaluated in this article, or claim that may be made by its manufacturer, is not guaranteed or endorsed by the publisher.
