# Supplementary material for: Substrate Stiffness Influences Structural and Functional Remodeling in Induced Pluripotent Stem Cell-Derived Cardiomyocytes
Source: Front Physiol. 2021 Aug 19;12:710619. doi: 10.3389/fphys.2021.710619 (PMC8416903; doi:10.3389/fphys.2021.710619)
Supplement: Supplementary file 1 [file Data_Sheet_1.PDF]

Supplemental File:

Substrate stiffness influences structural and functional remodeling in iPSC-derived cardiomyocytes

Arlene Körner[1,2], Matias Mosqueira[1], Markus Hecker[1,2], Nina D. Ullrich[1,2]*

[1]Heidelberg University, Institute of Physiology and Pathophysiology, Division of Cardiovascular Physiology, Im Neuenheimer Feld 307, 69120 Heidelberg, Germany;

[2]German Center for Cardiovascular Research (DZHK) partner site Heidelberg-Mannheim.

*Corresponding Author

Supplemental Tables:

Table S1: Statistical evaluation of the diastolic Ca^{2+} levels during spontaneous activity and pacing at 1 or 2 Hz in iPSC-CMs grown on different substrate stiffnesses; statistical significances are indicated for $p<0.05$ relative to glass.

**Basal Ca^{2+} levels (F_{340}/F_{360})**	**Glass**		**28 kPa**		**15 kPa**		**1.5 kPa**	
**spontaneous**	0.35±4*10^{-3}	n=30	0.36±0.01	n=20	0.36±0.01	n=26	0.36±0.01	n=16
**1 Hz**	0.36±0.01	n=27	0.40±0.01*	n=20	0.38±0.01	n=26	0.38±0.01	n=16
**2 Hz**	0.40±0.01	n=27	0.46±0.02*	n=20	0.43±0.01	n=26	0.43±0.01	n=16

Table S2: Summary of the EC-coupling data of I_{CaL} and Ca^{2+} transient amplitudes, EC-coupling gain at -25 mV and +10 mV in iPSC-CMs grown on different substrates; statistical significances are indicated for $p<0.05$ relative to glass.

EC-coupling		Glass	28 kPa	15 kPa	1.5 kPa
- 25 mV	$I_{Ca,L}$ (pA/pF)	-2.3±0.3 n=13	-2.6±0.4 n=14	-2.3±0.5 n=20	-1.7±0.2 n=16
	Ca^{2+} transient amplitude (F/F_0)	1.7±0.1 n=12	1.9±0.2 n=9	2.1±0.2 n=15	2.2±0.3 n=10
	ECC gain ($\Delta F/F_0$ / I_{CaL})	0.39±0.07 n=12	0.41±0.13 n=9	1.08±0.30* n=15	1.17±0.55* n=10
+ 10 mV	$I_{Ca,L}$ (pA/pF)	-6.33±0.9 n=15	-8.7±1.0 n=14	-8.1±0.9 n=20	-7.4±1.0 n=17
	tau of $I_{Ca,L}$ (ms)	19.6±2.9 n=11	19±3.1 n=11	17.2±2.8 n=12	18.8±1.9 n=12
	Ca^{2+} transient amplitude (F/F_0)	2.47±0.2 n=12	2.6±0.3 n=9	3.3±0.4 n=15	2.9±0.5 n=10
	ECC gain ($\Delta F/F_0$ / I_{CaL})	0.25±0.04 n=12	0.20±0.03 n=9	0.35±0.07 n=15	0.34±0.11 n=10

Table S3: Data summarizing steady-state Ca^{2+} transients and caffeine-induced Ca^{2+} transient amplitudes, fractional release and integrated NCX currents measured from iPSC-CMs grown on different surfaces; statistical significances are indicated for $p<0.05$ relative to glass.

	Glass		28 kPa		15 kPa		1.5 kPa	
Ca^{2+} transients ($\Delta F/F_0$)	1.26±0.20	n=10	1.16±0.31	n=5	1.68±0.25	n=8	1.29±0.49	n=9
Caffeine transients ($\Delta F/F_0$)	2.14±0.37	n=10	1.26±0.34	n=5	2.42±0.26	n=8	2.15±0.54	n=9
Fractional release (%)	64.2±6.8	n=10	92.3±2.4*	n=5	69.2±6.8	n=8	56.5±5.1	n=9
Integrated NCX (pC)	70.7±19.6	n=12	92.3±2.4	n=9	69.2±6.8	n=10	56.5±5.1	n=13

Table S4: Summarized data of combined cytosolic Ca^{2+} transients (fura-2) and contractions measurements and EC50 values of the Ca^{2+}-contraction loops from iPSC-CMs grown on different surfaces; statistical significances are indicated for $p<0.05$ relative to glass.

**Ca^{2+} transients**	**Glass** (n=30)	**28 kPa** (n=22)	**15 kPa** (n=26)	**1.5 kPa** (n=24)
**Amplitude ($\Delta F/F_0$)**	0.93±0.06	0.94±0.06	0.93±0.06	0.91±0.06
**TTP (ms)**	100.1±12.6	103.4±14.5	86.9±8.5	82.7±12.2
**FDHM (ms)**	221.2±22.9	233.2±27.5	213.8±20.9	190±22.2
**Decay (ms)**	186.5±15.7	226.7±19.4	203.8±13.6	180±13.8

**Shortening**	**Glass** (n=13)	**28 kPa** (n=14)	**15 kPa** (n=18)	**1.5 kPa** (n=23)
**Amplitude (%)**	3.6±0.3	4.6±0.6	4.6±0.4	9.6±0.7*
**TTP (ms)**	98.2±10.2	133.3±6.8	157.7±10.4*	197±18*
**FDHM (ms)**	124.3±13.1	198.7±12.2*	231.7±14.8*	275.7±19*
**Decay (ms)**	41.5±6	76.6±8.9	110.2±11.6*	196.8±12.7*

**Ca^{2+} loop**	**Glass** (n=13)	**28 kPa** (n=14)	**15 kPa** (n=16)	**1.5 kPa** (n=23)
**EC_{50} (F/F_0)**	1.37±0.04	1.27±0.03	1.23±0.02*	1.17±0.01*

Supplemental Figure 1:

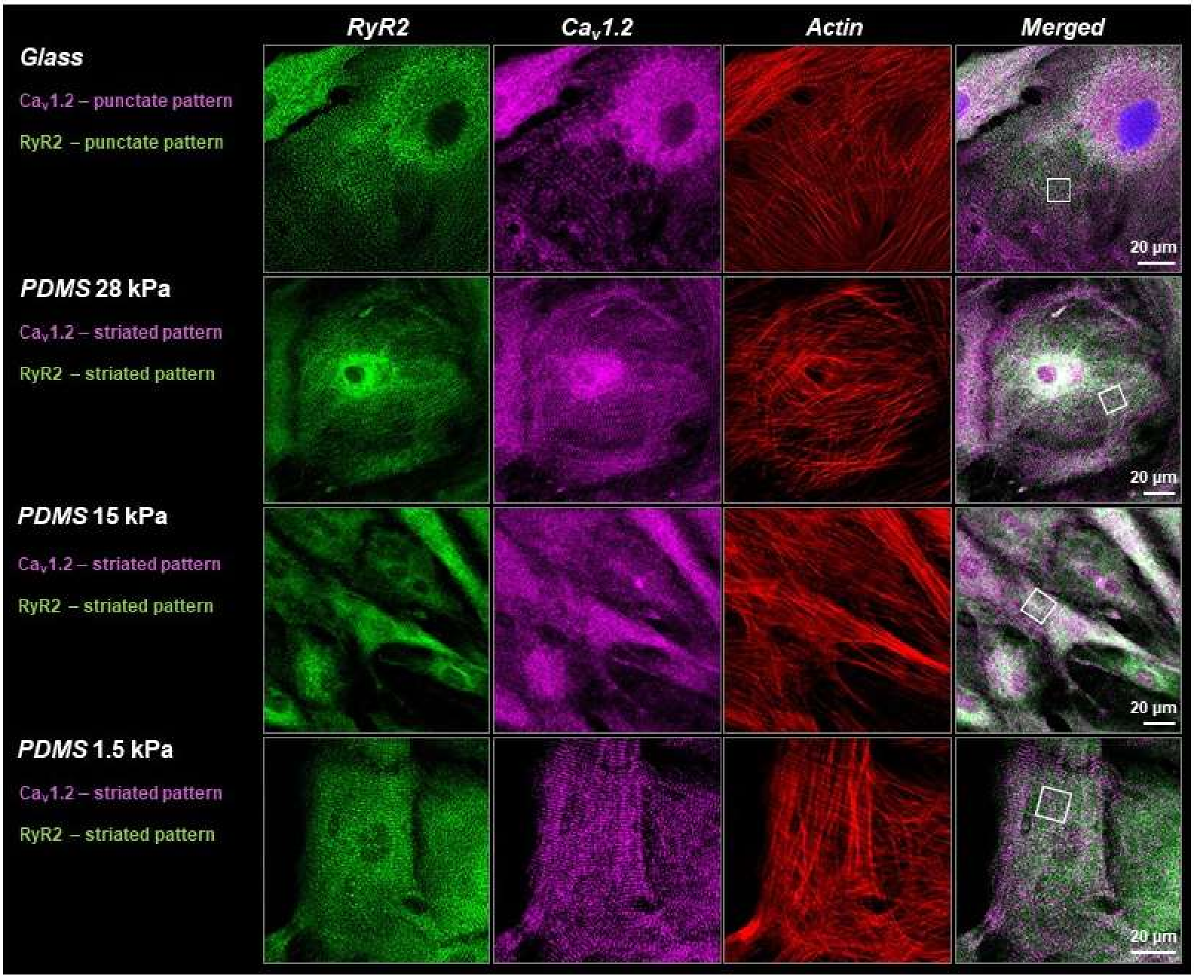

Suppl. Fig. 1: Overview images showing the expression pattern of Ca_v1.2 and RyR2 Ca^{2+} channels in iPSC-CMs grown on different culture surfaces. Shown are single stainings of RyR2 (green), Ca_v1.2 (purple), actin as reference for cardiomyocytes and merged images of RyR2 and Ca_v1.2 stainings. White boxes in the merged images indicate the detailed areas shown in Fig. 3 of this paper. The scale bar indicates 20 µm.

Supplemental Figure 2:

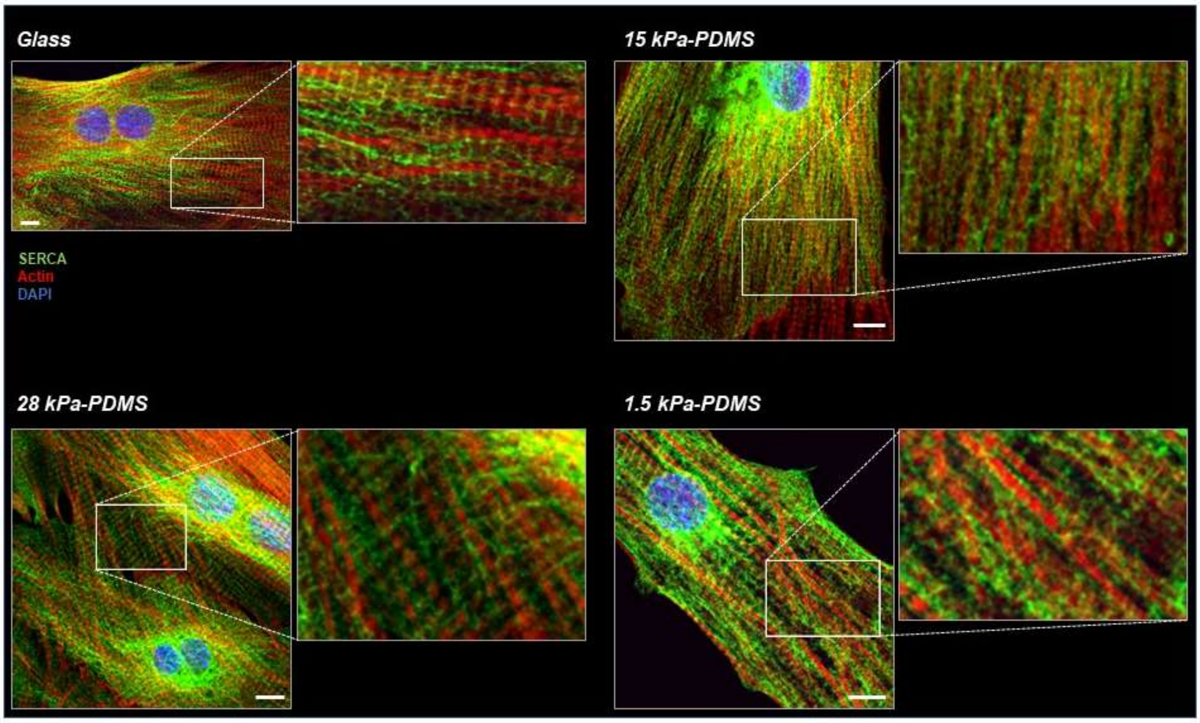

Suppl. Fig. 2: Overview and detailed images showing the expression pattern of SERCA in iPSC-CMs grown on different culture surfaces. Merged images show stainings of SERCA (green), actin (red) and DAPI for nuclear staining (blue). White boxes indicate the location of the detailed areas shown next to the overview images. The scale bar indicates 10 µm.

Supplemental Figure 3:

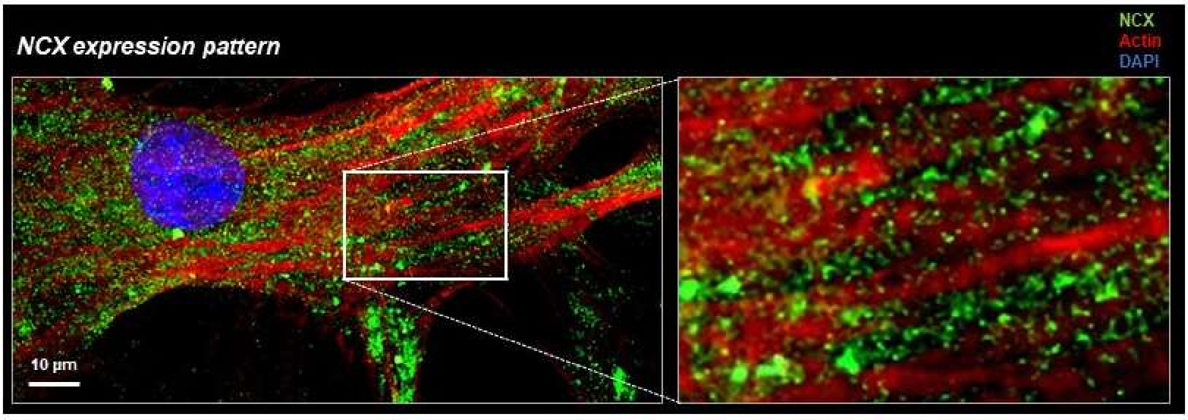

Suppl. Fig. 3: Overview and detail sample image showing the expression pattern of NCX in iPSC-CMs. Merged images show stainings of NCX (green), actin (red) and DAPI for nuclear staining (blue). The white box indicates the location of the detailed area shown on the right side. The scale bar indicates 10 µm.
